# Tongxinluo Protects against Hypertensive Kidney Injury in Spontaneously-Hypertensive Rats by Inhibiting Oxidative Stress and Activating Forkhead Box O1 Signaling

**DOI:** 10.1371/journal.pone.0145130

**Published:** 2015-12-16

**Authors:** Wei-min Luo, Jing Kong, Yan Gong, Xiao-qiong Liu, Rui-xue Yang, Yu-xia Zhao

**Affiliations:** 1 Key Laboratory of Cardiovascular Remodeling and Function Research, Qilu Hospital, Shandong University, Jinan, Shandong, China; 2 Department of Traditional Chinese Medicine, Qilu Hospital, Shandong University, Jinan, Shandong, China; 3 Department of Magnetic Resonance Imaging, Jinan hospital of infectious diseases, Jinan, Shandong, China; 4 Department of Cardiology, Qilu Hospital, Shandong University, Jinan, Shandong, China; The University of Manchester, UNITED KINGDOM

## Abstract

Hypertension is an independent risk factor for the progression of chronic renal failure, and oxidative stress plays a critical role in hypertensive renal damage. Forkbox O1(FoxO1) signaling protects cells against oxidative stress and may be a useful target for treating oxidative stress-induced hypertension. Tongxinluo is a traditional Chinese medicine with cardioprotective and renoprotective functions. Therefore, this study aimed to determine the effects of Tongxinluo in hypertensive renal damage in spontaneously hypertensive rats(SHRs)and elucidate the possible involvement of oxidative stress and FoxO1 signaling in its molecular mechanisms. SHRs treated with Tongxinluo for 12 weeks showed a reduction in systolic blood pressure. In addition to increasing creatinine clearance, Tongxinluo decreased urinary albumin excretion, oxidative stress injury markers including malondialdehyde and protein carbonyls, and expression of nicotinamide adenine dinucleotide phosphate oxidase subunits and its activity in SHR kidneys. While decreasing phosphorylation of FoxO1, Tongxinluo also inhibited the phosphorylation of extracellular signal-regulated kinase1/2 and p38 and enhanced manganese superoxide dismutase and catalase activities in SHR kidneys. Furthermore, histology revealed attenuation of glomerulosclerosis and renal podocyte injury, while Tongxinluo decreased the expression of α-smooth muscle actin, extracellular matrixprotein, transforming growth factor β1 and small mothers against decapentaplegic homolog 3,and improved tubulointerstitial fibrosis in SHR kidneys. Finally, Tongxinluo inhibited inflammatory cell infiltration as well as expression of tumor necrosis factor-α and interleukin-6. In conclusion, Tongxinluo protected SHRs against hypertension-induced renal injury by exerting antioxidant, antifibrotic, and anti-inflammatory activities. Moreover, the underlying mechanisms of these effects may involve inhibition of oxidative stress and functional activation of FoxO1 signaling.

## Introduction

In clinic practice, the hypertension-induced renal injury is an important factor in the pathogenesis of end-stage nephropathy and the need for dialysis[1]. Renal injury related to hypertension is characterized by glomerular and tubulointerstitial damages, which eventually lead to renal dysfunction[2].

Gradually increasing blood pressure and activation of the renin-angiotensin-aldosterone system are pro-oxidant and proinflammatory effects, and the initial factors contributing to renal damage[3, 4]. Oxidative stress plays a critical role in the pathological development of renal injury related to hypertension[5]. Reactive oxygen species (ROS) generated during oxidative stress influence nearly all types of intrinsic kidney cells. In hypertensive kidney damage, oxidative stress determines podocyte apoptosis and generation of segmental glomerulosclerosis, thereby influencing glomerular permeability[6]. Furthermore, oxidative stress also promotes the accumulation of myofibroblasts via epithelial-mesenchymal transition of proximal tubular and mesangial cells in the kidney, resulting in remodeling of the extracellular matrix of the tubulointerstitium[7]. In addition, oxidative stress and inflammatory responses act synergistically in the pathogenesis of renal injury[8]. Therefore, antioxidant therapy is an important aspect of the therapeutic strategy for hypertensive kidney damage[3, 9].

Among the various signaling pathways activated in response to oxidative stress, the forkhead box O1(FoxO1) transcription factor plays an important role in protecting cells. Under normal and pathological conditions,FoxO1 regulates the expression of specific antioxidant enzymes to protect cells against oxidative stress[10, 11].FoxO1 also inhibits epithelial-mesenchymal transition of mesangial cells and secretion of extracellular matrix(ECM) protein [12]. Furthermore, post-translational modifications control the function of FoxO1 protein[13, 14].

Tongxinluo (TXL)superfine powder, a traditional Chinese medicinal prescription, has been used clinically for 2 decades to treat a wide range of cardiovascular diseases including angina pectoris and hypertension[15, 16]. Experimental evidence has shown the pleiotropic effects of TXL in animals as well as its antioxidant, anti-inflammatory, and antifibrotic effects in subjects with cardiac and renal injury[17–19]. However, the therapeutic effects of TXL in hypertensive kidney damage have not been investigated. We hypothesized that TXL might protect against renal injury by regulating oxidative stress and FoxO1 signaling. To test this idea, we evaluated the effects of chronic treatment with TXL on renal structure and function in SHRs and further attempted to elucidate the possible mechanisms of action.

## Materials and Methods

### Ethics statement

The experiments conducted in this study conformed to the Animal Management Rule of the Chinese Ministry of Health (documentation 55, 2001), and the experimental animal protocol was approved by the Animal Care and Use Committee of Shandong University.

### Preparation of TXL ultrafine powder

TXL ultrafine powder was provided by Shijiazhuang Yiling Pharmaceutical Co., Ltd., (Hebei, China). Tongxinluo contains 12 medicinal components(Table 1).These materials were ground to a superfine powder(<10μm) using micronization technology after they were authenticated and standardized to marker compounds according to the 2010 Chinese Pharmacopoeia(National Pharmacopoeia Committee,2010). The TXL ultrafine powder was dissolved in normal saline and detailed preparation methods are described elsewhere [20].

**Table 1:** Formulation of Tongxinluo Ultrafine Powder.

Components	Voucher specimen number	Part used	Amount used (%)
Pamax giseng C.A.Mey.(extracion)	11,001	Root and rhizome	1.677
Paeonia lactiflora Pall.(extracion)	11,003	Root	1.558
Ziziphus jujuba Mill. Var. spimosa(Bunge) Hu ex H.F.Chou (extracion)	11,002	Seed	1.173
Santalum album L.(extracion)	11,004	Heartwood of stem	0.354
Dalbergia odorifera T.Chen(extracion)	11,005	Heartwood of stem and root	4.000
Steleophaga plancyi (Boleny) (Micro-oryzae farina)	12,003	Female dried body	18.111
Scolopendra subspinipes mutilans L. Koch (farina)	12,001	Dried body	3.623
Hirudo nipponica Whitman (farina)	12,004	Dried body	27.330
Cryptotympana pustulata Fabricius (farina)	12,005	Skin	18.111
Buthus martensii Karsch (farina)	12,002	Dried body	18.111
Boswellia carteri (farina)	11,006	Resin	5.927
Borneolum syntheticum (artificial)	11,007	C_10_H_18_O	3.623

### Animal protocol

Twenty 8-week-old male spontaneously hypertensive rats(SHRs, Vital River Animal Technique Corp., Ltd., Beijing, China) were randomly assigned to the untreated SHR and TXL groups (n = 10, each) while 10 male 8-week-old Wistar-Kyoto(WKY) rats (Experimental Animal Center of Shandong University, Jinan, China) were used as the normal control group. The TXL group was intragastrically administered TXL at 0.4 g·kg^-1^·day^-1^ while the untreated WKY and SHR groups were intragastrically administered equal volumes(1.5 mL) of saline once daily for 12 weeks. Body weight and systolic blood pressure (SBP) was assessed every 4 weeks throughout the study. The SBP was measured using the tail-cuff method in conscious rats after warming at 38°C for 10 min. The animals were treated for 12 weeks and then acclimated to the metabolic cages for 48 h before 24-h urine collection and subsequent measurement of albumin and creatinine content. The 24-h urine volume was measured, and then, the rats were anesthetized by intraperitoneal injections of pentobarbital, euthanized, and then blood samples were collected from the right ventricle to obtain the serum, which was stored at -80°C for subsequent analysis of creatinine concentrations. In addition, the kidneys were harvested, and a portion was fixed in 10% formaldehyde and embedded in paraffin before sectioning for histological analysis and immunohistochemical staining. Additional portions were snap-frozen in liquid nitrogen and stored at -80°C for western blot analysis and quantitative real-time polymerase chain reaction(qPCR) analysis.

### Measurements of renal functional parameters

Urinary albumin was quantified using a Nephrat II enzyme-linked immunosorbent assay (ELISA) kit(Exocell, Philadelphia, PA).Urinary and serum creatinine were quantified using a Quantichrom Creatinine Assay kit(BioAssay Systems, Hayward, CA) and creatinine clearance was calculated from these data and normalized to kidney weight as an index of glomerular filtration rate.

### Histological analysis

Renal tissues sections (4-μm thick)were stained with periodic acid-Schiff (PAS) and Masson trichrome staining for analysis of glomerulosclerosis and tubulointerstitial fibrosis. A semi-quantitative morphometric score index was used to evaluate the degree of glomerulosclerosis[21]. Sclerosis was defined as the collapse or obliteration of the glomerular capillary tuft or both, accompanied by hyaline material deposits or increase in the matrix, or both. The sclerotic severity for each glomerulus was graded from 0 to 4 as follows: 0, no lesions while 1, 2, 3, and 4 were <25,>25–50, >50–75, and>75–100% lesions, respectively. Thirty glomeruli were randomly selected in every five cross-sections per animal for morphometric analysis. The glomerular sclerotic index (GSI) was calculated using the following formula: GSI = (1× *n*_1_ + 2 × *n*_2_ + 3 × *n*_3_ + 4 × *n*_4_)/(*n*_0_ + *n*_1_ + *n*_2_ + *n*_3_ + *n*_4_), where *n*_*x*_ is the number of glomeruli observed in each grade of glomerulosclerosis. Tubulointerstitial fibrosis was assessed semi-quantitatively. Ten random high-power fields (400× magnification) per kidney were evaluated using the Image-Pro Plus 6.0 image analysis system to quantify the percentage of fibrosis in the area assessed.

### Western Blot analysis

Total protein was extracted from the kidney tissue samples using radioimmunoprecipitation lysis buffer (Beyotime, Shanghai, China) while cytoplasmic and nuclear protein fractions were extracted using a cytoplasmic and nuclear protein extraction reagent kit(Boster, Wuhan, China). Equal amounts of protein were separated using 10% sodium dodecyl sulfate-polyacrylamide gel electrophoresis (SDS-PAGE) and transferred to polyvinylidene fluoride (PVDF) membranes. After blocking with 5% non-fat milk for 1 h at room temperature, the membranes were incubated overnight at 4°Cwith one of the following primary antibodies: anti-FoxO1(1:1000,Cell Signaling Technology, Danvers,MA, USA), anti-phospho-FoxO1(1:500, Santa Cruz Biotechnology, CA,USA), anti-extracellular signal-regulated kinase(ERK)1/2 (1:1000), anti- phospho-ERK1/2(1:2000), anti-P38(1:1000), anti-phospho-P38(1:500), anti- phosphatidylinositol 3-kinase(PI3K,1:1000), anti-phospho-PI3K(1:1000), anti-Akt (1:2000), anti-phospho-Akt(1:1000), anti-adenosine monophosphate-activated protein kinase(AMPK,1:2000), anti-phospho-AMPK(1:1000), anti-SIRT1(1:5000), anti-transfoming growth factor β1(TGFβ1,1:500),anti-small mothers against decapentaplegic homolog 3(SMAD3, 1:1000), anti-phospho-SMAD3 (1:2000, Abcam,Cambridge,UK), and β-actin (1:500, Zhongshan Goldenbridge, Beijing, China). Next, the membranes were incubated with horseradish peroxidase (HRP)-conjugated secondary antibodies (Zhongshan Goldenbridge, Beijing, China) at room temperature for 1 h. The protein bands were visualized using a FluorChem E data system (Cell Biosciences, San Jose, CA, USA) and quantified by densitometry using the Image J Software (National Institutes of Health, NIH, Bethesda, MD, USA).

### Quantitative Real-Time Polymerase Chain Reaction

Total RNA was extracted from the kidney tissue samples with TRIzol (Invitrogen, Carlsbad, CA, USA) and reverse-transcribed using a complementary DNA reverse transcription kit (Takara Bio, Tokyo, Japan). Reactions were performed in an iQ5 real-time PCR thermocycler (Bio-Rad, Hercules, CA, USA) using SYBR green as the fluorescent dye. The mRNA expression of the target genes was normalized to the control gene, glyceraldehyde 3-phosphate dehydrogenase (GAPDH) using the comparative threshold cycle (2^-ΔΔCT^) method. S1 Table lists the primer sequences used in the real-time PCR experiments.

### Immunohistochemical staining

Formalin-fixed (10%) 4-μmkidney tissue sections were immunostained as follows. Briefly, the sections were deparaffinized, washed with phosphate-buffered saline (PBS),incubated with 3% hydrogen peroxide (H_2_O_2_)in methanol to block the endogenous peroxidase activity, and then treated with an antigen unmasking solution consisting of 10 mmol/L sodium citrate (pH 6.0) and 0.05% Tween 20. Nonspecific binding was blocked with 5% normal goat serum in PBS. Then, the sections were incubated overnight withanti-α-SMA, anti-desmin, anti-fibronectin(1:500 each, Abcam, Cambridge, UK), anti-collagen IV,and anti-cluster of differentiation (CD) 68 (1:200, Santa Cruz Biotechnology, CA, USA)primary antibodies in a humidified chamber at 4°C. The first antibody was visualized using an indirect immunoperoxidase method and digital images were obtained using an Olympus DP72 microscope(Tokyo, Japan).We analyzed 10 randomly selected frames(400× magnification) using the Image Pro Plus 6.0(Media Cybernetics, Houston, TX,USA).

### Nicotinamide Adenine Dinucleotide Phosphate oxidase activity

Nicotinamide adenine dinucleotide phosphate (NADPH) oxidase activity was measured in kidney tissue homogenates at room temperature in an assay mixture consisting of 50 mM phosphate buffer(pH 7.1), 0.01 mM ethylenediaminetetraacetic acid (EDTA), and 250 μM lucigenin. The tissue homogenate was first added to the reaction mixture to attain an equilibrium (3–5 min) with no chemiluminescence, and then the reaction was started by the addition of 100 μM NADPH. Chemiluminescence generated in response to NADPH was recorded over a period of 3 min in the presence or absence of 25 μM diphenyliodonium, an inhibitor of NADPH oxidase (NOX). The diphenyliodonium-inhibited activity wasconsidered the NOX activity and expressed as the emitted relative light units (RLU)·s^-1^·mg protein^-1^.

### Malondialdehyde and protein carbonyls levels and catalase and superoxide dismutase activities

The malondialdehyde(MDA)and protein carbonyls levels as well as catalase and superoxide dismutase(SOD) activities in renal tissue were measured using assay kits purchased from Nanjing Jiancheng Bioengineering Institute (Nanjing, China)according to manufacturers' instructions as described previously[22].In brief, MDA, protein carbonyls, catalase, and SOD were detected using the thiobarbituric acid, ammonium molybdate with H_2_O_2_, and xanthine oxidase methods, respectively. The absorbance of the assay reactions was spectrometrically measured at 532, 370,405, and 550 nm, respectively. The protein concentrations of all the renal tissue homogenate samples were determined using the bicinchoninic acid assay (BCA) method.

### Statistical analysis

Data are expressed as mean±standard error of the mean (SEM). Differences between groups were analyzed using a one-way analysis of variance (ANOVA) followed by Student-Newman-Keuls post hoc test. Repeated-measures data were analyzed using a two-way ANOVA and ordinal data were analyzed using Kruskal-Wallis test. All statistical analyses were performed using the statistical package for the social sciences(SPSS) software version 18.0(SPSS, IBM Corp., Armonk, NY, USA).Differences were considered statistically significant at P<0.05.

## Results

### General data

There was no significant difference in body weight, kidney weight, and urinary volume among the three groups (Table 2).

**Table 2:** Summary of general data after 12-week treatment.

General data	WKY	SHR	TXL
**Body weight(g)**	320±9	318+8	316±9
**Kidney weiht(g)**	1.3±0.2	1.4±0.3	1.3±0.3
**Water intake(ml/24 h)**	30.8±2.5	28±1.9	31.2±2.2
**Urinary volume(ml/24 h)**	35.2±2.2	30.3±1.8	34±2

Data are expressed as mean±SEM; n = 10 rats per group.WKY, Wistar Kyoto; SHR, spontaneously hypertensive rat.

#### TXL decreased SBP of SHRs

**Fig 1:**
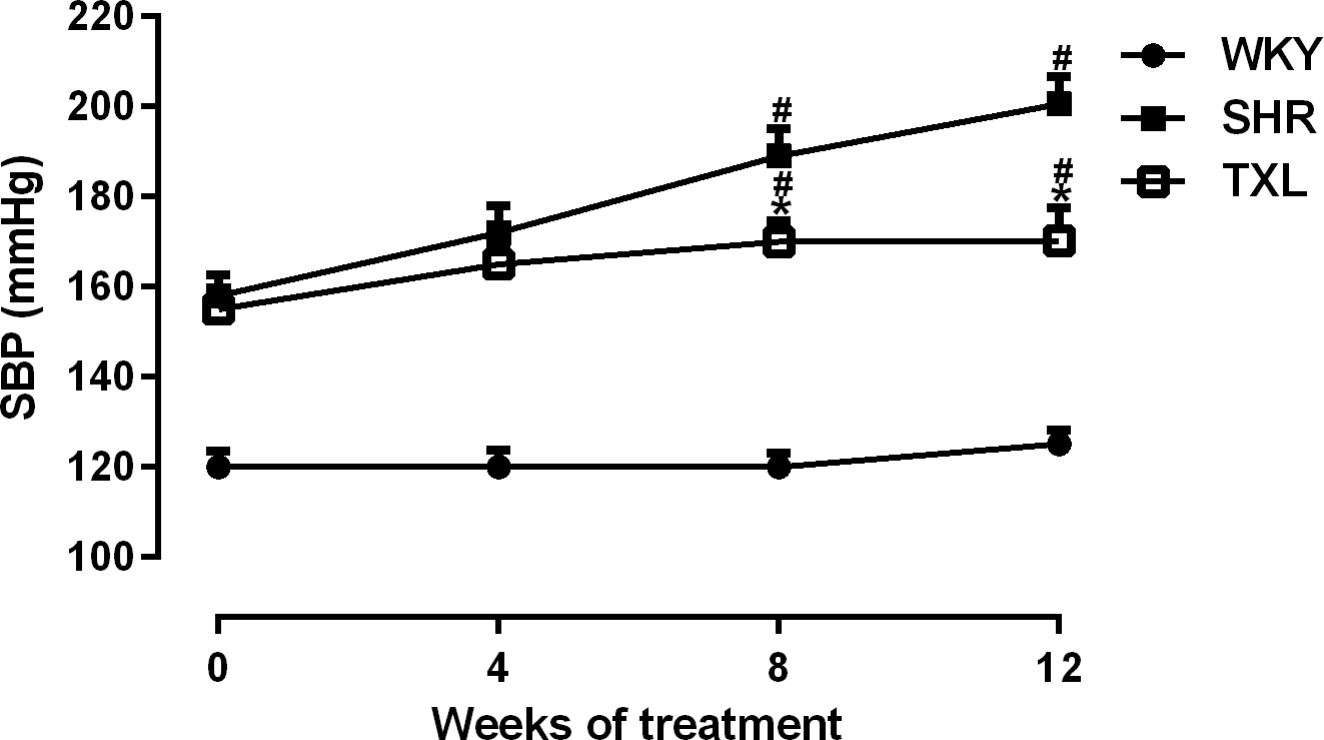
Effect of Tongxinluo (TXL) on systolic blood pressure (SBP). Data are expressed as mean±SEM; n = 10 rats per group, ^#^P < 0.05 and ^*^P < 0.05 vs. WKY and SHR groups, respectively. WKY, Wistar Kyoto; SHR, spontaneously hypertensive rat.

The SBP of SHRs gradually increased with age and was significantly higher than that of the WKY controls was(P <0.05,WKY vs. SHR and TXL). However, chronic treatment with TXL significantly inhibited that trend and reduced the SBP by 15% compared with that of the untreated SHR group by the end of the 12-week treatment(P<0.05,TXL vs. SHR, Fig 1).

#### TXL attenuated renal functional injury in SHRs

**Fig 2:**
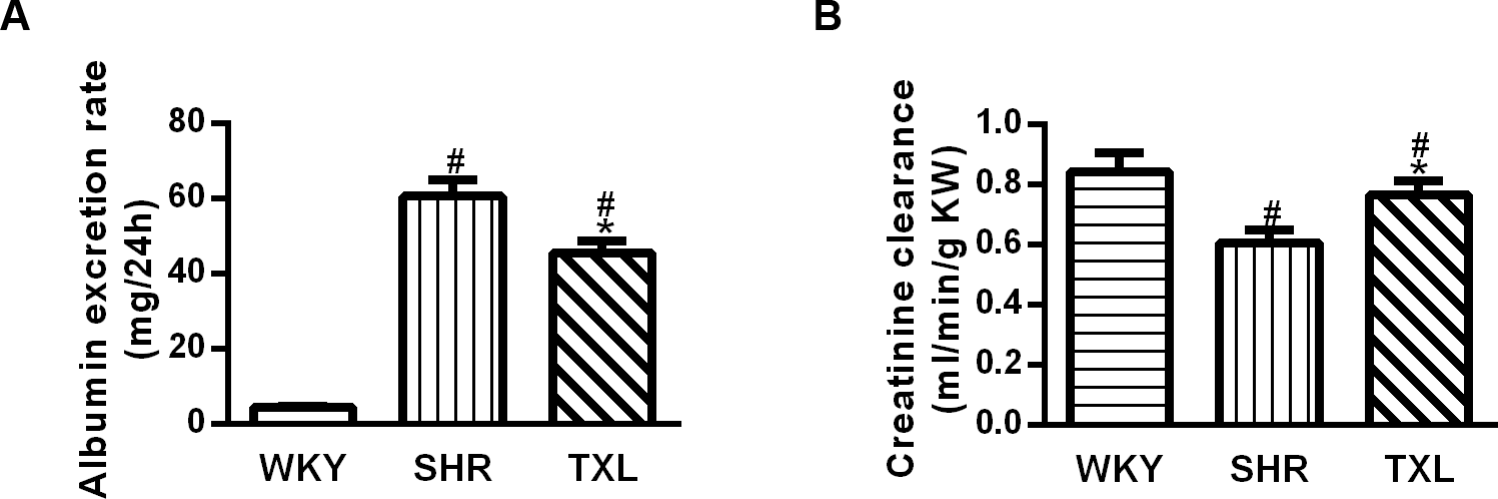
Functional evaluation of effects of Tongxinluo (TXL)in hypertensive kidney injury. Quantitative data of (A) urinary albumin excretion rate and (B)creatinine clearance. Data are expressed as mean±SEM; n = 10 rats per group, ^#^P<0.05 and^*^P<0.05, vs. WKY and SHR groups, respectively. WKY, Wistar-Kyoto; SHR, spontaneously hypertensive rat.

Renal function was estimated by urine albumin excretion rate and creatinine clearance. Compared with the WKY rats, the SHRs showed an increased urine albumin excretion rate and reduced creatinine clearance (P <0.05,WKY vs. SHR and TXL) while TXL treatment significantly decreased the urine albumin excretion rate and increased creatinine clearance(P <0.05,TXL vs. SHR, Fig 2A and 2B).

#### TXL inhibited oxidative stress injury in SHR kidneys

**Fig 3:**
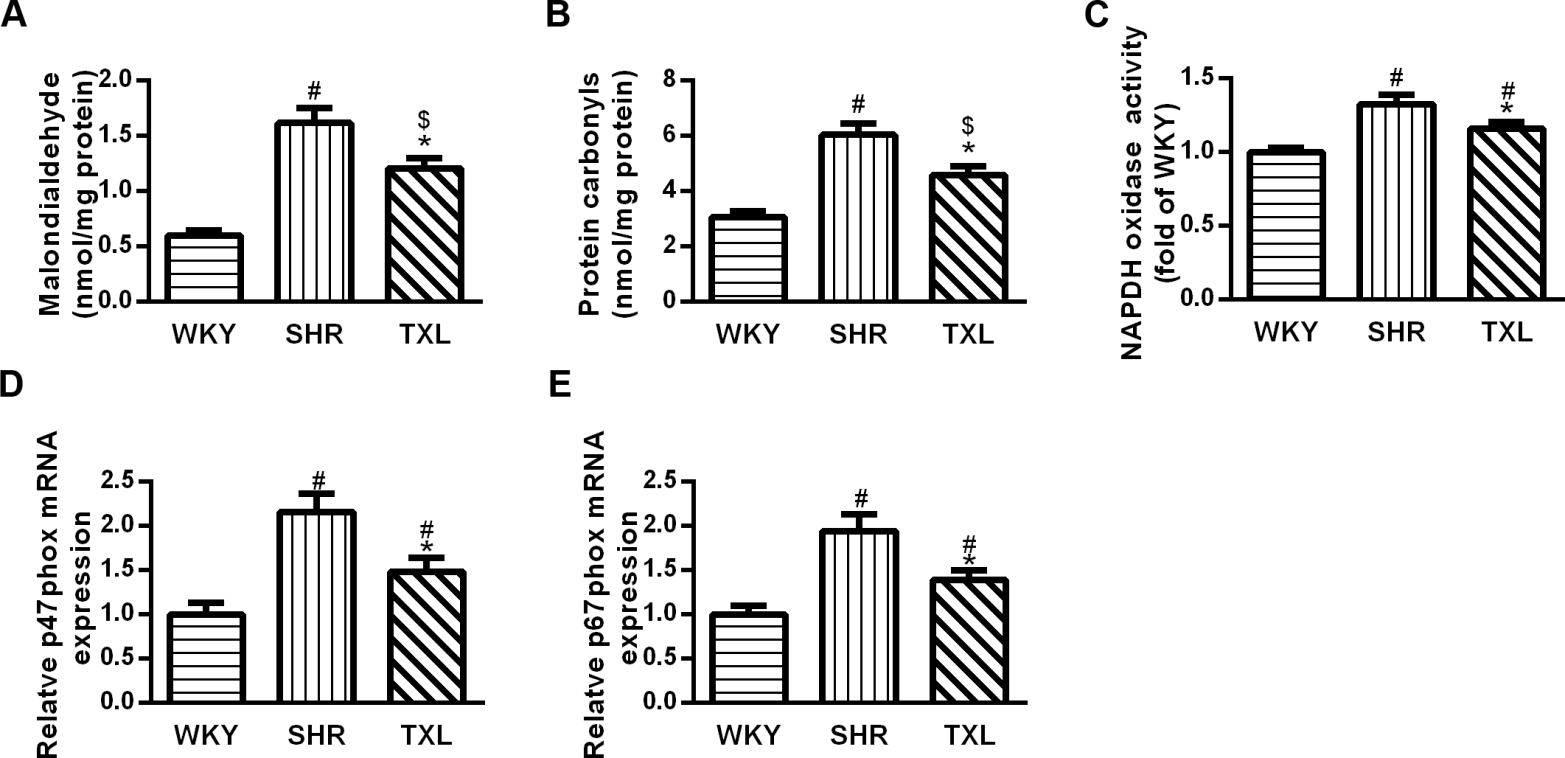
Tongxinluo (TXL) inhibited oxidative stress in spontaneously hypertensive rat (SHR) kidneys. Quantitative analysis of (A)MDA level and (B)protein carbonyls. (C) Quantitative analysis of NAPDH oxidase (NOX). PCR analysis of and (D) p47phox and (E) p67phox. Data are mean±SEM; n = 10 rats per group, ^#^P<0.05 and ^$^P>0.05 vs. WKY group, respectively;^*^P<0.05 vs. the SHR group. MDA, malondialdehyde; NAPDH, nicotinamide adenine dinucleotide phosphate; PCR, polymerase chain reaction; WKY, Wistar-Kyoto.

Oxidative stress injury was evaluated by determining the levels of related biomarkers including MDA and protein carbonyls in the kidney, which significantly increased in the SHR group compared to the WKY group(P <0.05,WKY vs. SHR).However, treatment with TXL significantly decreased the levels of both markers in the SHR kidneys(P <0.05,TXL vs. SHR) and made them not significantly different from the WKY controls(P >0.05, TXL vs. WKY, Fig 3A and 3B).

Furthermore, NOX mediates the production of oxidative free radicals. The expression of NOX subunits p47phox and p67phox, as well as NOX activities significantly increased in the SHR groups compared to that in the WKY group(P <0.05,WKY vs. SHR and TXL). However, this effect significantly decreased following TXL treatment(P <0.05,TXL vs. SHR, Fig 3C–3E).

#### TXL promoted activation of FoxO1 in SHR kidneys

**Fig 4:**
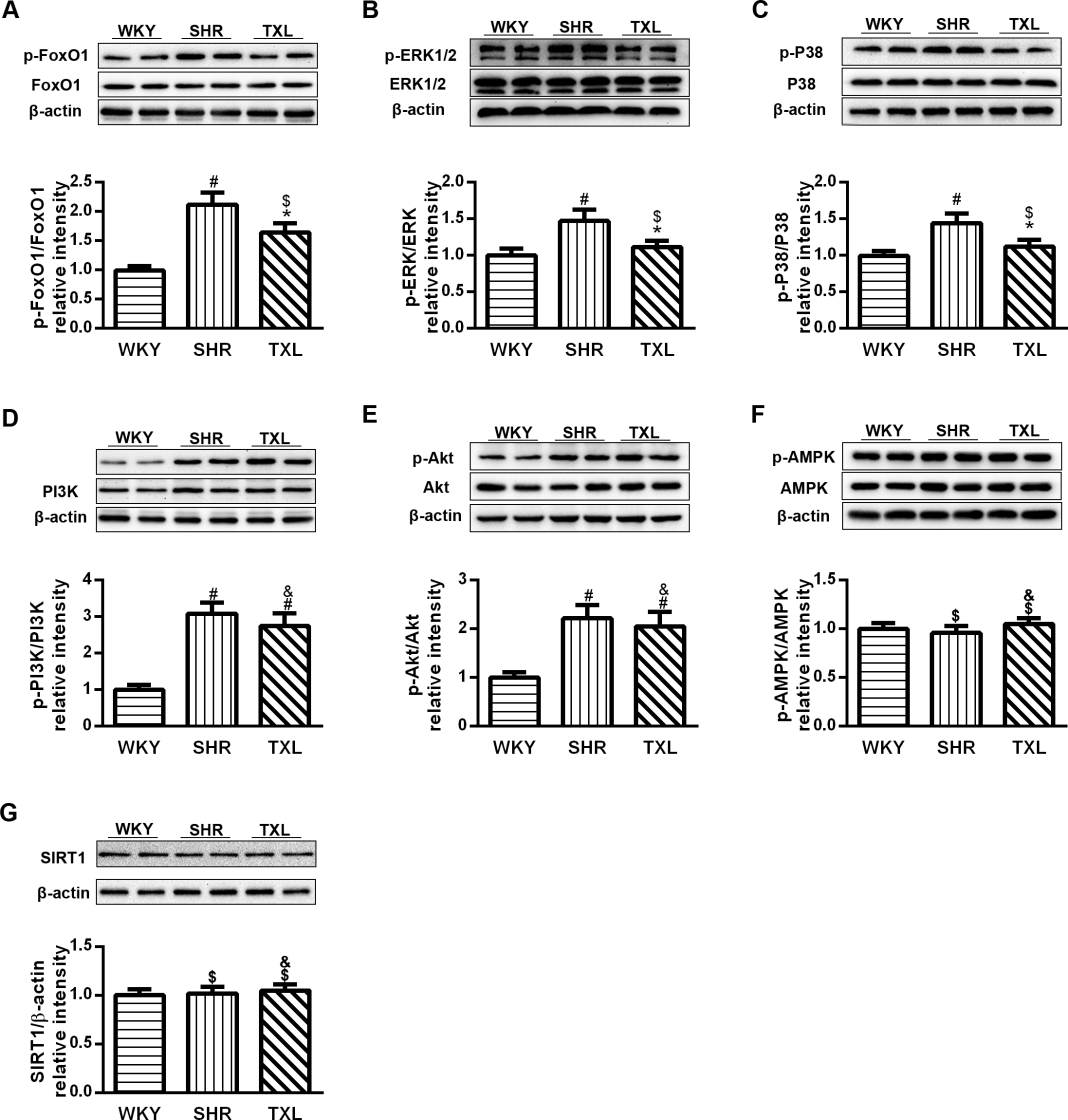
Tongxinluo (TXL) altered forkhead box O1 (FoxO1) signaling in spontaneously hypertensive rat (SHR) kidneys. Western blot analysis of (A) FoxO1 and phospho-FoxO1, (B) ERK1/2 and phospho-ERK1/2, (C) p38 and phospho-p38, (D) PI3K and phosphor-PI3K, and (E) Akt and phosphor-Akt (F) AMPK and phosphor-AMPK, and (G)SIRT1 after 12 weeks of TXL treatment. Data are mean±SEM; n = 10 rats per group, ^#^P<0.05 and ^$^P>0.05 vs. WKY group, respectively; ^*^P<0.05 and ^&^P>0.05 vs. SHR group, respectively. ERK, extracellular signal-regulated kinase; PI3K, phosphatidylinositol 3-kinase;AMPK, Adenosine monophosphate-activated protein kinase; WKY, Wistar-Kyoto.

We used western blot analysis to investigate the effect of TXL on the activation of FoxO1 signaling and determined the ratio of phosphorylated FoxO1. The results revealed that FoxO1 phosphorylation was significantly higher in the SHR group than it was in the WKY group(P <0.05,WKY vs. SHR). The phosphorylation of FoxO1 in the TXL group was significantly downregulated after 12 weeks of treatment (P <0.05,TXL vs. SHR), and was not significantly different from that in the WKY controls. (P>0.05, TXL vs. WKY, Fig 4A).

As the functional activation of FoxO1 depends on post-translational modification which involves a series of signaling pathways, we observed the changes of signaling molecules including MAPK, PI3K, Akt, AMPK and SIRT1. Firstly, we measured the phosphorylation of MAPK pathway molecules including ERK1/2 and p38, which was significantly enhanced in the SHR group compared with that in the WKY control group(P <0.05,WKY vs. SHR). However, phosphorylation of ERK1/2 and p38 was significantly downregulated in the SHRs (P <0.05,TXL vs. SHR) after treatment with TXL for 12 weeks and was not significantly different from that in the WKY group(P >0.05, TXL vs. WKY, Fig 4B and 4C). Secondly, compared with the WKY controls, the phosphorylation of PI3K and Akt was significantly enhanced in SHR kidneys(P<0.05,WKY vs. SHR and TXL) and this effect was not significantly decreased after treatment with TXL in SHRs(P >0.05, TXL vs. SHR, Fig 4D and 4E). Thirdly, there was no significant difference in the renal expression of SIRT1 or phosphorylation of AMPK between the WKY and SHR groups(P >0.05, WKY vs. SHR), while treatment with TXL had no significant effects on these proteins in SHR kidneys(P >0.05, TXL vs. SHR, Fig 4F and 4G).

#### TXL promoted antioxidant activity in SHR kidneys

**Fig 5:**
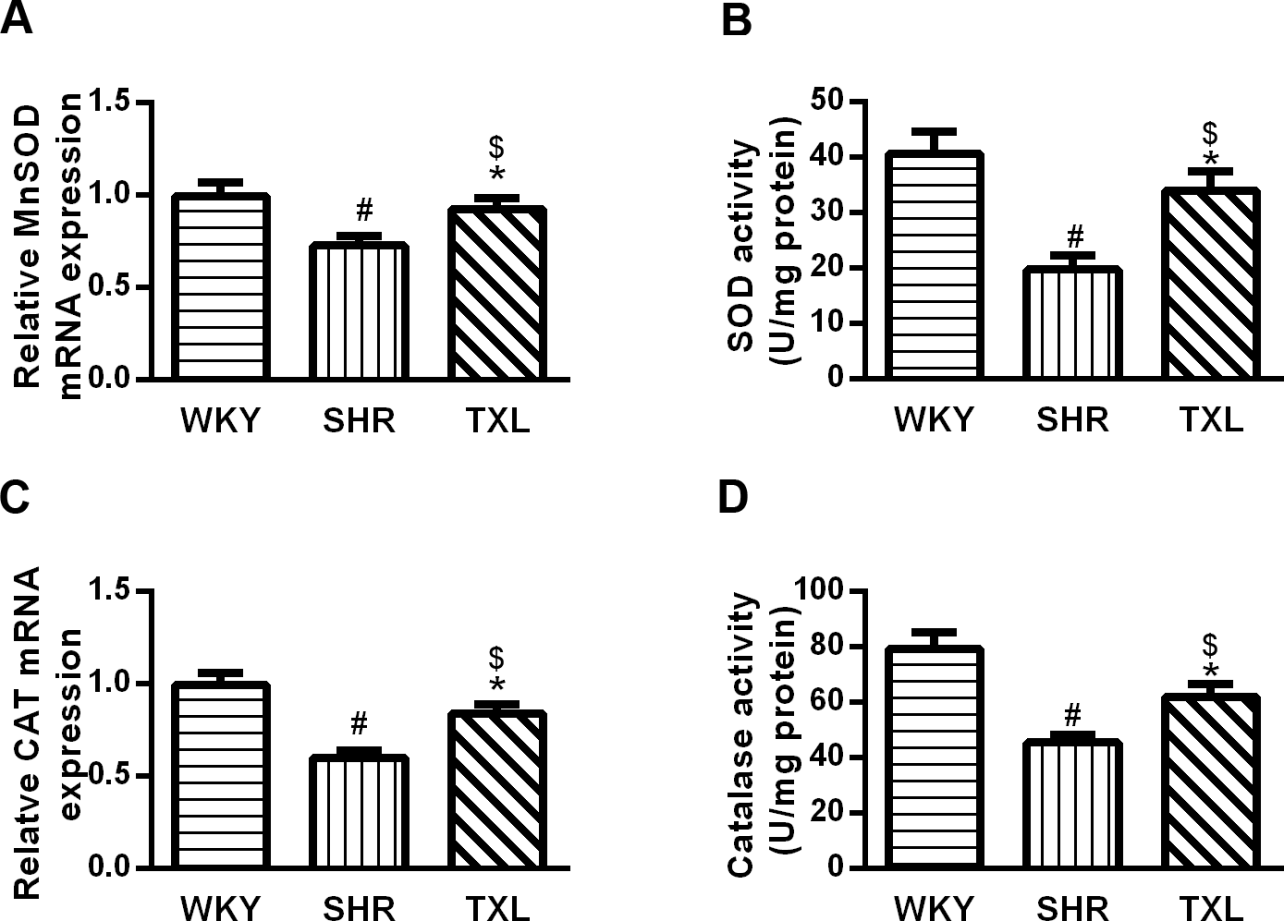
Effects of Tongxinluo (TXL) on rat kidney antioxidant activities. Quantitative analysis of MnSOD (A) PCR analysis and (B)SOD activities. Quantitative analysis of catalase (C) PCR analysis and (D)activities. Data are mean±SEM; n = 10 rats per group, ^#^P<0.05 and ^$^P>0.05, vs. WKY group, respectively;^*^P<0.05 vs. SHR group. MnSOD, manganese superoxide dismutase; PCR, polymerase chain reaction; WKY, Wistar-Kyoto; SHR, spontaneously hypertensive rat.

**Fig 6:**
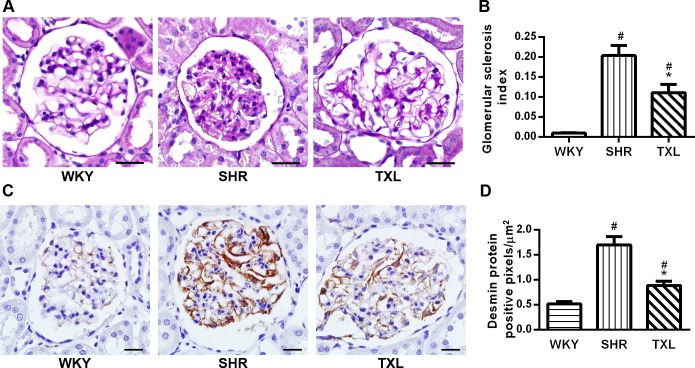
Effects of Tongxinluo (TXL) on rat glomerular injury in hypertensive kidney injury. Representative images of (A)PAS of kidney tissue sections. Scale bar, 50 μm. Quantitative analysis of (B) glomerular sclerosis index. (C)Representative images and (D)Quantitative analysis of desmin immunostaining. Scale bar, 20 μm. Data are expressed as mean±SEM; n = 10 rats per group, ^#^P < 0.05 and^*^P < 0.05 vs. WKY and SHR groups, respectively. PAS, periodic acid-Schiff; WKY, Wistar-Kyoto; SHR, spontaneously hypertensive rat.

The antioxidant function of the rat kidneys was assessed by determining the expression and activities of manganese (Mn)SOD and catalase, which are FoxO1 target genes. The expression and activities of these genes were significantly lower in the SHR group than they were in the WKY group(P <0.05,WKY vs. SHR), and the levels were significantly increased in SHR kidneys after TXL treatment(P <0.05,TXL vs. SHR). There wae no significant difference on the expression and activities of MnSOD and catalase between the TXL group and the WKY group(P >0.05,TXL vs. WKY, Figs 5A–6D).

#### TXL prevented glomerular injury in SHR kidneys

The effects of TXL on the glomerular structural injury were evaluated using PAS, which showed that segmental glomerulosclerosis was present only in a few glomeruli in the SHR group and nearly absent in the WKY group. The glomerular sclerosis index for the SHRs was significantly higher than that of the WKY rats was(P <0.05,WKY vs. SHR and TXL); however, it was significantly reduced following treatment with TXL(P<0.05,TXL vs. SHR, Fig 6A and 6B).

We evaluated the effects of TXL on podocyte injury using desmin immunostaining. The results showed that compared with the WKY controls, the SHRs showed significantly renal higher expression of desmin, which indicated injury to the podocytes(P<0.05,WKY vs. SHR and TXL). However, TXL treatment attenuated this expression in the kidneys of SHRs(P<0.05,TXL vs. SHR, Fig 6C and 6D).

**Fig 7:**
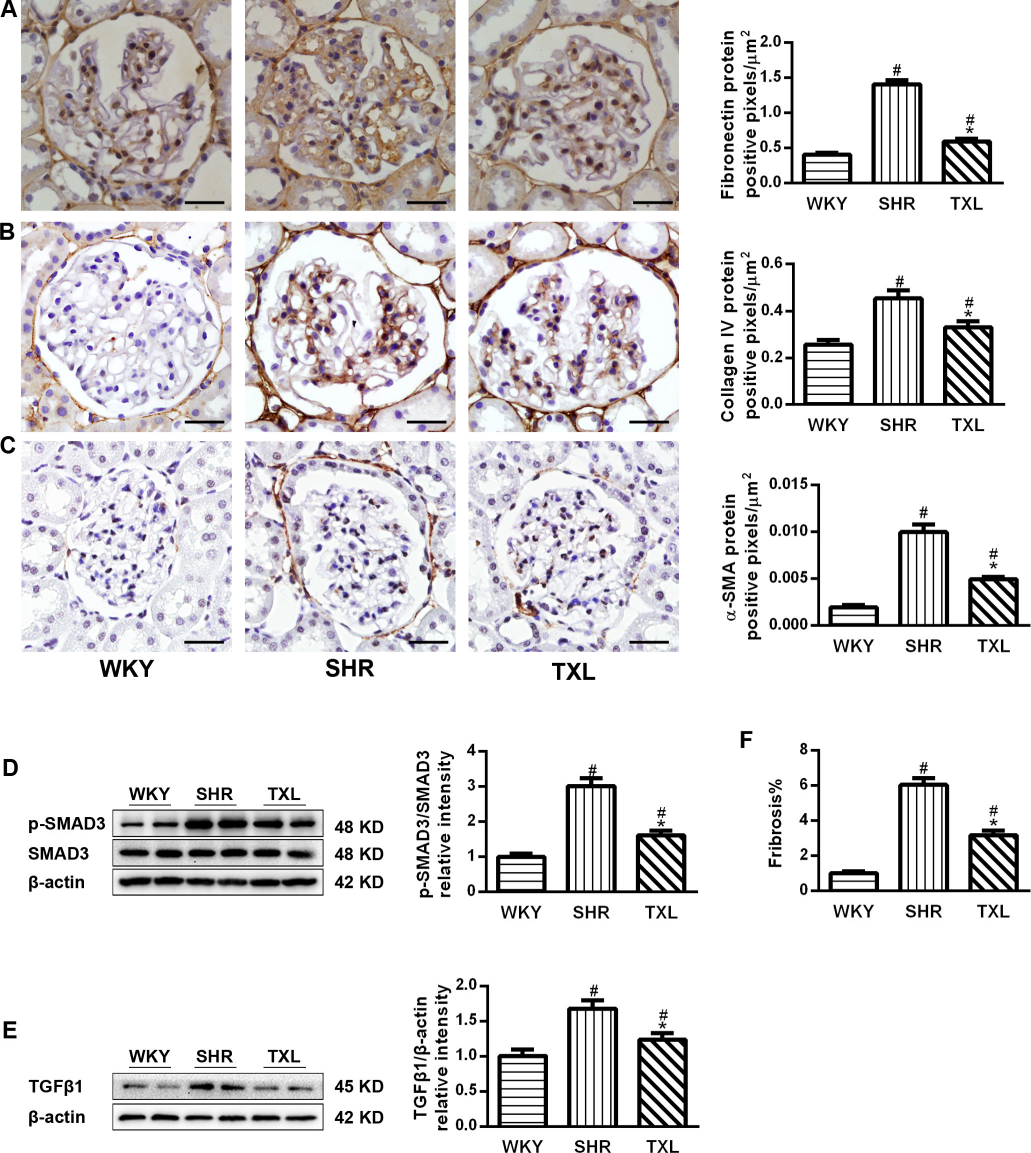
Effects of Tongxinluo(TXL) on fibrotic mediators in spontaneously hypertensive rat(SHR) kidneys. Representative images and quantitative analysis of(A) α-SMA,(B)collagen IV and (C) fibronectin immunostaining. PCR analysis of(D)TGFβ1 and (E) SMAD3.(F)Quantitative analysis of tubulointerstitial fibrosis. Scale bar, 20 μm. Data are mean±SEM; n = 10 rats per group, ^#^P<0.05 and^*^P<0.05 vs. WKY and SHR groups, respectively. α-SMA, α-smooth muscle actin; PCR, polymerase chain reaction; TGFβ1,transforming growth factor β1;SMAD3, small mothers against decapentaplegic homolog 3;WKY, Wistar-Kyoto.

TXL decreased fibrotic mediators and renal fibrosis in SHR kidneys. α-SMA expression is the major feature of activated myofibroblasts in the kidney while fibronectin and collagen IV are abnormally deposited in hypertension-induced nephropathy. Transforming growth factor β1(TGFβ1)/SMAD3 signaling plays a key role in epithelial-mesenchymal transition (EMT) and fibroblast activation. Therefore, we determined the expression levels of α-SMA, fibronectin and collagen IV, which increased significantly in the SHR groups compared with the WKY group(P <0.05,WKY vs. SHR and TXL). Moreover, the levels of these proteins were significantly lower in the TXL-treated group than they were in the untreated SHR group (P <0.05,TXL vs. SHR, Fig 7A–7C). In addition, the expression of TGFβ1 and the phosphorylation of SMAD3 significantly increased in the SHR groups compared with that of the WKY group(P<0.05,WKY vs. SHR and TXL), while TXL treatment significantly reduced the expression(P <0.05,TXL vs. SHR, Fig 7D and 7E).

Furthermore, we analyzed tubulointerstitial fibrosis in SHRs, and the Masson’s trichrome staining showed that it was significantly higher in the SHRs than it was in the WKY group(P <0.05,WKY vs. SHR and TXL). Moreover, the TXL-treated SHRs showed a decrease in tubulointerstitial fibrosis compared to the degree of fibrosis in the untreated SHRs(P<0.05,TXL vs. SHR, Fig 7F).

#### TXL inhibited inflammatory responses in SHR kidneys

**Fig 8:**
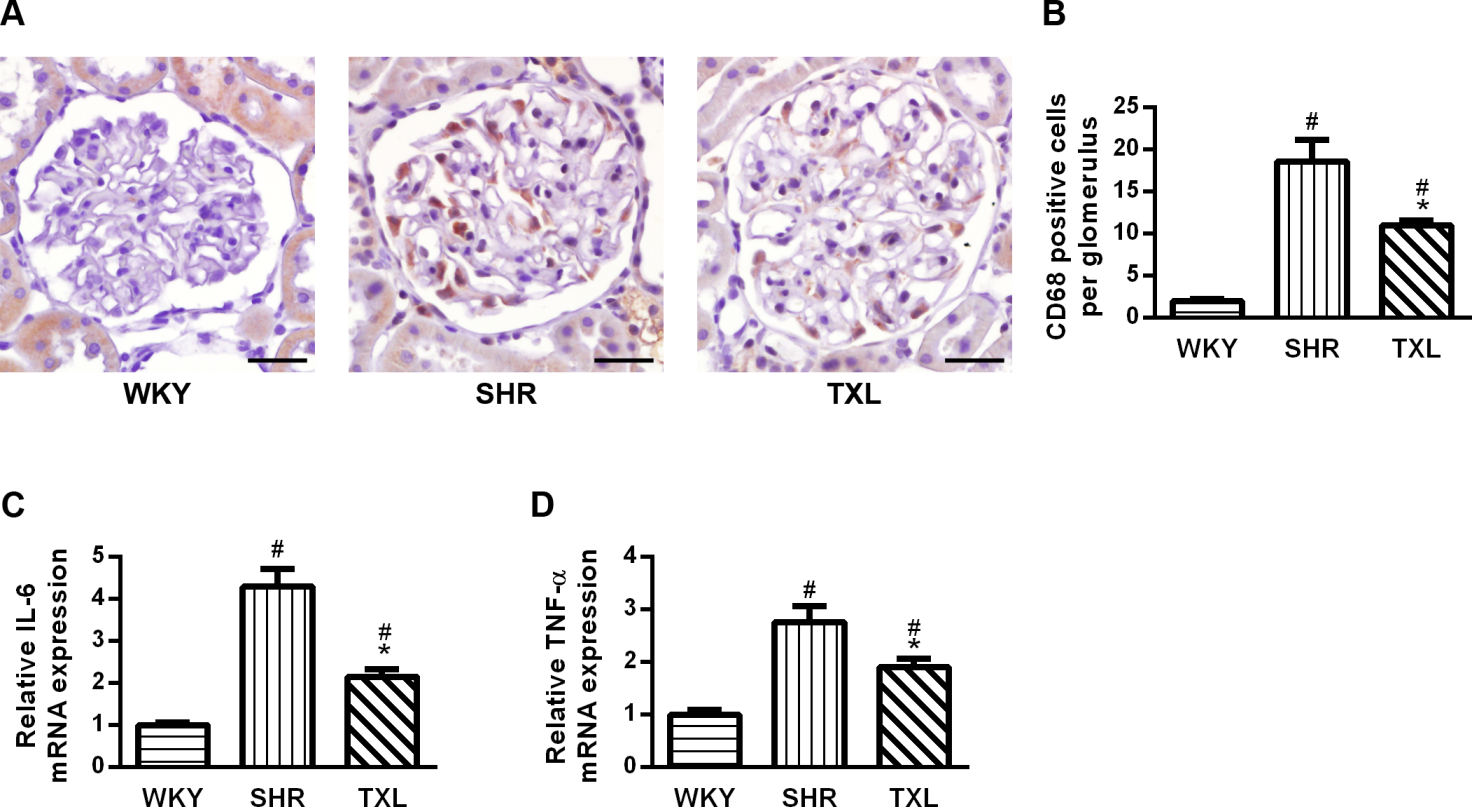
Effects of Tongxinluo (TXL) on inflammatory responses in spontaneously hypertensive rat(SHR) kidneys. (A)Representative images and (B)quantitative analysis of CD68 immunostaining; scale bar, 20 μm.PCR analysis of (C) TNF-α and(D) IL-6. Data are mean±SEM; n = 10 rats per group, ^#^P<0.05 and ^*^P<0.05vs.WKY and SHR groups, respectively.CD68, cluster of differentiation 68; PCR, polymerase chain reaction; TNF, tumor necrosis factor; IL, interleukin; WKY, Wistar-Kyoto.

To evaluate the effects of TXL on inflammatory responses in SHR kidneys, we investigated the expression of CD68 and inflammatory mediators. CD68 immunopositivity and the expression of tumor necrosis factor (TNF)-α and interleukin (IL)-6 were higher in the kidney sections of both SHR groups than they were in the WKY group(P <0.05,WKY vs. SHR and TXL).However, the level was significantly lower in the TXL-treated group than it was in the untreated SHR group (P <0.05,TXL vs. SHR, Fig 8A–8D).

## Discussion

TXL superfine power is a compound formulated based on the meridian theory of traditional Chinese medicine. It is a mixture of plant and insect products following a strict proportion for each and was approved for clinic use by the State Food and Drug Administration of China in 1996(S2 Table). In our laboratory, we first provided information on the constituents of TXL using high-performance liquid chromatography (HPLC) for fingerprint analysis of its aqueous extract[20].Furthermore, pharmaceutical analysis has demonstrated that ginsenoside Rg1,ginsenoside Rb1,peoniflorin, jujuboside A, and jujuboside B are the five most important active components in TXL. Due to its multiple ingredients, TXL shows pleiotropic effects that are potentially of therapeutic value. Experimental evidence showed that TXL protected against renal structural and functional injury in diabetic nephropathy[19]. Ginsenoside Rg1 protected podocytes against oxidative stress as well as autophagic and complement-mediated injury[23, 24].Ginsenoside Rg1 inhibited renal interstitial fibrosis by reducing the expression of TSP-1, promoting the repair of the peritubular capillary, and suppressing the process ofepithelial-to-mesenchymal transition via the phosphorylation of ERK1/2[25, 26]. Ginsenoside Rb1prevented intestinal ischemia-reperfusion-induced injury in kidneys and ameliorated oxidative damage and renal interstitial fibrosis in rats with unilateral ureteral obstruction[27, 28]. Paeoniflorin prevented renal interstitial fibrosis partly by blocking EMT and exerted a nephroprotective effect by the inhibition of macrophage infiltration[29, 30].

This study demonstrated for the first time that TXL treatment protected SHRs against hypertensive renal injury by reducing the SBP, decreasing urinary albumin excretion, increasing creatinine clearance, and improving glomerulosclerosis and tubulointerstitial fibrosis. Furthermore, we found that TXL diminished the level of MDA and protein carbonyls, decreased renal expression of fibrotic mediators including α-SMA,TGFβ1,SMAD3,fibronectin and collagen IV, and inflammatory mediators including IL-6 and TNF-α.TXL also prevent podocyte injury, epithelial-mesenchymal transition, and inflammatory cell infiltration, as well as inhibited the phosphorylation of FoxO1 and MAPK, thereby restored the antioxidant abilities of the kidney.

Clinically, essential hypertension is a common pathogenic factor for renal damage, and persistent and gradually increasing blood pressure is the main initiator of injury to the renal structure and function[1]. The SHR is a commonly used model of established hypertension, which has similarities to the human form such as a pre-hypertensive phase, development and sustenance of hypertensive phases, and end-organ damages[31]. In our study, TXL treatment progressively reduced the SBP in SHRs, and effective control of blood pressure may contribute to protecting against renal injury, which is consistent with the actions of other antihypertensive medications [32, 33].

Our results revealed that renal injury in SHRs is characterized by glomerulosclerosis, tubulointerstitial fibrosis, and proteinuria, which is consistent with previous studies performed in this model[33, 34].Proteinuria often occurs before renal dysfunction in hypertension, which indirectly verifies the presence of structural injury to the kidney, and becomes a causative factor in renal damage progression[34]. Therefore, the reduction of urinary protein excretion after TXL treatment demonstrated the possibility of an early protective effect against hypertensive renal injury. Furthermore, creatinine clearance is regarded as a reliable indicator of glomerular filtration rate. The higher creatinine clearance in the TXL-treated group than in the untreated SHR group provided evidence that TXL exerts its renoprotection even in the absence of evidence of renal dysfunction during the progression of hypertension.

Abnormal hemodynamic and neurohumoral changes in hypertension lead to renal oxidative stress and inflammation [3]. The kidney is vulnerable to oxidative stress, which is regarded as a vital factor in the progression of renal injury in different animal models of hypertension[5]. In addition, oxidative stress extensively affects major types of kidney cells.[5].Furthermore, studies have shown that pre-hypertensive SHRs from 2–3weeks old exhibit elevated renal oxidative stress compared to that exhibited by age-matched WKY rats[35]. Therefore, to determine the effects of TXL on oxidative stress in SHRs, we first evaluated markers of oxidative stress injury. The levels of MDA, a prominent marker for the assessment of lipid peroxidation and protein carbonyls, a production of oxidation or oxidative cleavage of proteins, significantly increased in SHR kidneys compared to that of the WKY rat kidneys, corroborating previous reports[21, 35, 36].Furthermore, chronic treatment with TXL decreased the levels of MDA and protein carbonyls, which indicated an improvement in oxidative stress injury. Then, we assessed the expression of NOX subunits p47phox and p67phox and NOX activity, which mediates the production of reactive oxygen species (ROS). TXL reduced renal p47phox and p67phox expression and NAPDH oxidase activity compared to that in untreated SHRs. These antioxidant effects are consistent with our previous study in pressure overload-induced heart failure[17].

Among the series of intracellular signals that mediate the renal oxidative stress process, FoxO1 signaling plays an important role in protecting cells against oxidative stress. Increased cellular oxidative stress mainly promotes FoxO1 phosphorylation, which results in its translocation from the nucleus to the cytoplasm, leading to the inhibition of target gene transcription[13, 22]. In our study, FoxO1 phosphorylation was upregulated in SHR kidneys, while TXL treatment reduced this effect, thereby it was retained in the nuclei and promoted the expression of target genes, which exerted antioxidant activities.

The functions of FoxO1 are controlled by different post-translational modifications such as phosphorylation, acetylation, and ubiquitylation[13]. Therefore, we investigated the most critical pathway. First, the MAPK family members including ERK1/2 and p38partially participate in the phosphorylation and functional regulation of FoxO1[14].It has been reported that oxidative stress activates the MAPK pathway and the renal injury induced is associated with an increase in ERK1/2 and p38 in different models of hypertension[37].TXL treatment downregulated phosphorylation of ERK1/2 and p38 in SHR kidneys, which was consistent with previous studies[18]. Second, in response to exposure to oxidative stress the phosphoinositide 3-kinase (PI3/K) pathway is activated and is another regulator of FOXO1 activity[38]. The results showed the phosphorylation of PI3K/AKT is upregulated in SHR kidneys compared with the WKY controls, and TXL did not alter the level of the phosphorylated PI3K/AKT signaling. In addition, AMPK modulates the activities of FoxO1 by either directly phosphorylating FoxO1 or altering the deacetylating effect of SIRT1 on FoxO1; furthermore, SIRT1 can activate the FoxO1 selectively and enhance the nuclear trapping and the expression of target genes of FoxO1[38].There was no significant difference between the WKY and TXL-treated rats in the phosphorylated AMPK, and SIRT1, suggesting that TXL treatment failed to alter these proteins. The PI3K pathway is an important cellular survival pathway and AMPK and SIRT1 control the energetic metabolism while MAPKs tend to modulate the stress and inflammatory responses [13]. TXL appeared to tend to improve the inflammation and antioxidative stress, consistent with other studies on the anti-inflammation and antioxidant activities despite affecting different signaling pathways [17, 18, 20].

It is well known that FoxO1 plays an important role in protecting cells against oxidative stress because it upregulates the gene expression of several antioxidant enzymes such as MnSOD and catalase[39].These enzymesprotect tissues from oxidative stress damage by catalyzing the conversion of ROS. Consistent with a previous study[21], we found that the expressions of MnSOD and catalase, as well as their activity levels, were decreased in SHR kidneys while TXL treatment enhanced these effects. Therefore, the antioxidant activity of TXL may largely depend on its regulation of FoxO1.

Activated FoxO1 participates in diverse cellular responses to oxidative stress in the glomerulus, tubulointerstitium, and different types of renal cells [13, 22]. First, we investigated the effects of TXL on the glomerular injury. In our study, the histological analysis showed that TXL treatment attenuated glomerular sclerosis in SHRs. Especially, we investigated renal podocytes, which are highly specialized cells that are located adjacent to the glomerular capillaries and form part of the glomerular filtration barrier. Furthermore, the loss of podocytes leads to proteinuria[40]while oxidative stress determines podocyte apoptosis and depletion in segmental glomerular sclerosis[6]. In our study, the number of injured podocytes(desmin positive) in the SHRs was significantly higher than it was in the age-matched WKY rats while TXL treatment decreased this number. Therefore, the antioxidant activity of TXL may have protected the podocytes against injury.

Second, oxidative stress also promotes the accumulation of myofibroblasts via epithelial-mesenchymal transition of proximal tubular and mesangial cells in the kidney, leading to interstitial fibrosis. Myofibroblasts, identified by positive α-SMA staining, are characterized by excessive secretion of profibrotic factors and ECM proteins[7, 12, 41].Activated FoxO1 suppresses the transcription of target genes of SMAD3 by antioxidant actions, which inhibit its activity[12]. TGFβ1/SMAD3 signaling is a vital factor governing the EMT, myofibroblast activation, and expression of ECM protein[42]. In our study, we determined the expression of major ECM proteins including FN, collagen IV, and α-SMA, which were increased in SHR kidneys, consistent with the activation of SMAD3signaling. TXL reduced the expression of ECM proteins and interfered with SMAD3 signaling, similar to its effects in the kidneys of rats with diabetic nephropathy[19]. Therefore, the effects of TXL on antioxidant activity and FoxO1 modulation may contribute to the improvement of interstitial fibrosis.

Third, oxidative stress and inflammatory responses act synergistically in mediating hypertension-related renal injury. Oxidative stress leads to inflammatory cell infiltration in the kidney[43], and loss of functional FoxO1 may lead to inflammatory cellactivation [44]. However, dephosphorylated and activated FoxO1 may enhance inflammation by increasing the expression of several proinflammatory genes[45]. In this study, CD68 immunopositive macrophages confirmed there was as an elevation in inflammatory cells, and the expression of inflammatory mediator including TNF-α and IL-6 were increased significantly, consistent with a previous study[46]. Although TXL promoted FoxO1 signaling, it still decreased inflammatory cell infiltration and inhibited inflammatory factor expression in the SHR kidneys. Except for the alleviation of oxidative stress, we propose that TXL exerted its anti-inflammatory effects via numerous other pathways simultaneously. Oxidative stress also activates ERK1/2 and p38 MAPK in SHR kidneys, which promotes the phosphorylation of FoxO1 and inhibits its antioxidant function[14]. ERK1/2 and p38 MAPK activation is greatly increased in intrinsic and infiltrating cells and regulates the transduction and production of inflammatory mediators in nephropathy[47]. Our results showed that TXL inhibited the activation of ERK1/2 and p38 MAPK. On one hand, TXL promoted the antioxidant activities via the activation of FoxO1 while it inhibited the expression of inflammatory mediators related to the activation of ERK1/2 and p38 MAPK. However, we cannot ignore the fact that the inhibitory effects of TXL on other inflammatory factors, such as nuclear factor kappa B, may participate in the anti-inflammatory effects, as shown in other studies [18, 20].Additionally, the functions of FoxO1 are also controlled by other post-transcriptional modulations such as acetylation and ubiquitylation[13], and the effects of TXL on these modulations still need to be studied further.

## Conclusion

To the best of our knowledge, this study is the first to provide mechanistic evidence that TXL protects against renal injury related to hypertension. Moreover, TXL exerts antioxidant, antifibrotic, and anti-inflammatory effects in hypertensive renal injury. Furthermore, the inhibition of oxidative stress and regulation of FoxO1 signaling were likely involved in its mechanism. This study is clinically significant because it demonstrates that TXL may be an alternative and complementary pharmacological approach to improving hypertension as well as preserving renal function and structure in patients who are predisposed to hypertension.

However, it should be noted that there are some limitation to the use of TXL. Firstly, although TXL ameliorated renal structural and functional injury in part, it failed to achieve normalization of all parameters. The combination of TXL with other drugs such as antihypertensive agents may contribute to improving the therapeutic effects and the synergistic effects require further investigation. Secondly, although the aqueous extract or solution of TXL was widely used in most of the experiments in our studies and in other’s research at present, it remains unclear that whether all the compounds found in TXL have to be present in these precise proportions to produce the observed effects or whether they are due to the presence of a single ingredient. Therefore, the active components of TXL and their interactions should be further elucidated in future. Finally, in pathological conditions related to hypertensive renal injury, there are multitudinous cell types including intrinsic and infiltrating cells and several associated signaling pathways. Therefore, further studies are required to elucidate the detailed and specific mechanisms underlying the actions of TXL to provide further evidence to support its therapeutic potential in hypertensive therapy.

## Supporting Information

S1 Table Primer pair sequences used for the real-time PCR analysis. (DOCX)

S2 Table Tongxinluo(TXL) Has no Effect on Physiological Parameters of WKY Rats. (DOCX)
