# Supplementary material for: Tongxinluo Protects against Hypertensive Kidney Injury in Spontaneously-Hypertensive Rats by Inhibiting Oxidative Stress and Activating Forkhead Box O1 Signaling
Source: PLoS One. 2015 Dec 16;10(12):e0145130. doi: 10.1371/journal.pone.0145130 (PMC4686063; doi:10.1371/journal.pone.0145130)
Supplement: S1 Table — (DOCX) [file pone.0145130.s001.docx]

**S1 Table. Primer pair sequences used for the real-time PCR analysis**

**.**

| Target | Forward | Reverse |
| --- | --- | --- |
| p47phox | TCACCGAGATCTACGAGTTC | TCCCATGAGGCTGTTGAAGT |
| p67phox | CAGTTCAAGCTGTTTGCCTG | TTCTTGGCCAGCTGAGCCAC |
| Catalase | ATGAAGCAGTGGAAGGAGCA | TCA AAGTGTGCCATCTCGTC |
| MnSOD | ACCGAGGAGAAGTACCACGA | TAGGGCTCAGGTTTGTCCAG |
| TNFα | TACTGAACTTCGGGGTGATTGGTCC | CAGCCTTGTCCCTTGAAGAGAACC |
| IL-6 | CCAATTTCCAATGCTCTCCT | ACCACAGTGAGGAATGTCCA |
| GAPDH | TGTGTCCGTCGTGGATCTGA | TTGCTGTTGAAGTCGCAGGAG |

*GAPDH*, glyceraldehyde-3-phosphate dehydrogenase; *MnSOD*, manganese superoxide dismutase; *TNF-α*, tumor necrosis factor; *IL-6*, interleukin-6.
