# Supplementary material for: Tongxinluo Protects against Hypertensive Kidney Injury in Spontaneously-Hypertensive Rats by Inhibiting Oxidative Stress and Activating Forkhead Box O1 Signaling
Source: PLoS One. 2015 Dec 16;10(12):e0145130. doi: 10.1371/journal.pone.0145130 (PMC4686063; doi:10.1371/journal.pone.0145130)
Supplement: S2 Table — (DOCX) [file pone.0145130.s002.docx]

**S2 Table. Tongxinluo(TXL) Has no Effect on Physiological Parameters of WKY Rats**

| Parameters | WKY group(n=10) | TXL-treated WKY group(n=5) |
| --- | --- | --- |
| Body weight(g) | 320±9 | 310±9 |
| Systolic blood pressure (mmHg) | 125±3 | 124±4 |
| Urinary albumin rate(mg/24 h) | 4.43±0.32 | 4.60±0.4 |
| Creatinine clearance(mL/min) | 0.84±0.06 | 0.86±0.08 |
| Glomerular sclerotic index | 0.01±0.0003 | 0.01±0.0002 |

Data are expressed as mean±SEM. WKY, Wistar Kyoto.
